# Surgical treatment of temporal lobe epilepsy: comparative results of selective amygdalohippocampectomy versus anterior temporal lobectomy from a referral center in Brazil

**DOI:** 10.1055/s-0043-1771172

**Published:** 2023-07-26

**Authors:** Laryssa Crystinne Azevedo Almeida, Vanessa Alves Lobato, Maria do Carmo Vasconcelos Santos, Aline Curcio de Moraes, Bruno Silva Costa

**Affiliations:** 1 Santa Casa de Misericórdia de Belo Horizonte, Departamento de Neurologia e Neurocirurgia, Belo Horizonte MG, Brazil.

**Keywords:** Epilepsy, Temporal Lobe, Hippocampal Sclerosis, Anterior Temporal Lobectomy, Epilepsies, Partial, Epilepsia do Lobo Temporal, Esclerose Hipocampal, Lobectomia Temporal Anterior, Epilepsias Parciais

## Abstract

**Background**  Temporal lobe epilepsy (TLE) is a high prevalence neurological disorder. Surgery has emerged as a promising treatment.

**Objective**  The objective of this work is to compare the surgical results of anterior temporal lobectomy (ATL) versus selective amygdalohippocampectomy (SAH) in a cohort of 132 patients.

**Methods**  We performed a retrospective study of 146 patients operated for TLE from 2008 to 2019. Initially, 13 patients were excluded from the study due to insufficient medical record data or follow-up loss. One patient was excluded from the analysis of the results due to death in the first postoperative week. We used the ILAE scale to classify seizure control after surgery. In patients with left hippocampal sclerosis, SAH was performed and in right temporal lobe epilepsy, ATL was the approach of choice.

**Results**  The mean follow-up time after surgery was 57.2 months (12–137). In our data analysis, we found that the group of patients undergoing ATL had a higher prevalence of being completely seizure-free (ILAE I) (57.1% versus 31%) and a higher rate of satisfactory seizure control (88.6% versus 69.3%) *p*  = 0,006, when compared with patients undergoing SAH.

**Conclusion**  The literature is still controversial about seizure control concerning the technique used due to the lack of a robust methodology. Our data analysis identified the superiority of ATL over SAH in seizure outcomes. ATL may be the best option for adequately controlling seizures with minimal additional morbidity in countries with a cost limitation for extended propaedeutics.

## Resumo

**Antecedentes**  A epilepsia do lobo temporal (TLE) é uma desordem neurológica de alta prevalência. A cirurgia surgiu como um tratamento promissor.

**Objetivo**  O objetivo deste trabalho é comparar os resultados da lobectomia temporal anterior (ATL) versus amigdalohipocampectomia seletiva (SAH) em uma coorte de 132 pacientes.

**Métodos**  Realizamos um estudo retrospectivo de 146 pacientes operados por TLE de 2008 a 2019. Inicialmente, 13 pacientes foram excluídos por insuficiência de dados em prontuário ou perda de seguimento. Um paciente foi excluído da análise por óbito na primeira semana de pós-operatório. Usamos a escala ILAE para classificar o controle das crises após a cirurgia. Em pacientes com esclerose hipocampal à esquerda, foi realizada a SAH, e na epilepsia do lobo temporal à direita, a ATL foi a abordagem de escolha.

**Resultados**  O tempo médio de seguimento após a cirurgia foi de 57,2 meses (12–137). Em nossa avaliação, encontramos que o grupo de pacientes submetidos à ATL apresentou maior prevalência de ausência total de crises (ILAE I) (57,1% versus 31%) e maior taxa de controle satisfatório da epilepsia (88,6% versus 69,3%) *p*  = 0,006, quando comparado ao grupo submetido à SAH.

**Conclusão**  A literatura ainda é controversa em relação à redução das crises de acordo com a técnica utilizada devido a falta de uma metodologia robusta. Nosso estudo identificou superioridade da ATL sobre a SAH nos desfechos convulsivos. ATL pode ser a melhor opção para controlar adequadamente as convulsões com morbidade adicional mínima em países com limitação de custo para propedêutica estendida.

## INTRODUCTION

 Temporal lobe epilepsy (TLE) is the most common human epileptic syndrome 1 being a disabling and progressive entity. 2 In addition to seizures, which already represent clinical management difficulties, they may also be associated with cognitive, language, or psychiatric disorders. 3 4 A tendency to drug refractoriness characterizes it, and up to a third of patients are drug-resistant. 5 In recent years, surgery has proven to be a therapeutic option with good results, with a controlled clinical trial demonstrating its superiority to drug treatment alone. 6

 The hippocampal sclerosis etiology is multifactorial, typically caused by inflammatory, infectious insults, trauma, or febrile seizures. 7 8

 There are technical variations in TLE surgery, and there is no consensus on the best surgical approach. The most common techniques are Anterior temporal lobectomy (ATL) and selective amygdalohipocampectomy (SAH). Foerster pioneered subtotal temporal lobectomy in 1925. 9 Falconer developed en bloc resection of the temporal lobe and mesial structures in 1953. 10 Morris, in 1956 used the term standard temporal lobectomy for a 6.5 cm resection of the temporal lobe, 11 12 Spencer refined this technique with 4.5 cm in the non-dominant cortex and 3 cm in the dominant cortex. Niemeyer, in 1958, described a transventricular selective access to mesial temporal structures through an incision in the medial temporal lobe. 13 Wieser and Yasargil proposed a transsilvian approach to the amygdala and hippocampus. 14

 There is still controversy about the best surgical approach for mesial temporal epilepsy. 15 Elseways, selective resections of mesial structures could have less cognitive effects, whereas an anterior temporal lobectomy has better seizure control. 

 In a review in 2008, Schramm 16 cites eight studies that compared selective surgery against temporal lobectomy concerning seizure control. In six of these studies, the authors found no difference in seizure control despite the surgical approach. ATL was more effective in two papers, one in children. 17 18 Josephson 19 compared ATL and SAH in a meta-analysis of 13 articles and 1203 patients, showing better control of seizures in ATL. 

## METHODS

### Ethical statement

All patients signed an informed consent, and the study was conducted following the Declaration of Helsinki. The national ethics board approved the study.

### Participants and evaluation

 A retrospective study was performed based on the medical records of 146 patients operated on for temporal lobe epilepsy from 2008 to 2019. The ILAE classification 20 ( Table 1 ) was used to determine the degree of seizure control, and we compared the descriptive results according to the technique used. 

**Table 1:** ILAE outcome classification

Outcome classification	Definition
1	Completely seizure free; no auras
2	Only auras; no other seizures
3	One to three seizure days per year; ± auras
4	Four seizure days per year to 50% reduction of baseline seizure days; ± auras
5	Less than 50% reduction of baseline seizure days to 100% increase of baseline seizure days; ± auras
6	More than 100% increase of baseline seizure days; ± auras

 The preoperative evaluation of these patients included neuropsychological testing, video-EEG, and high-resolution MRI. In cases where the video-EEG with scalp electrodes failed to define the temporal lobe as an epileptogenic source, a foramen ovale electrode was implanted as a complementary method. We included only patients with unilateral hippocampal sclerosis on MRI and concordant epileptic onset on video-EEG. Non-invasive options such as functional MRI would add additional costs and time, being a method not exempt from clinical differences. 21 Postoperatively, an MRI was requested for all patients to assess whether the resection area was satisfactory.

### Approach and selection of groups

**Figure 1:**
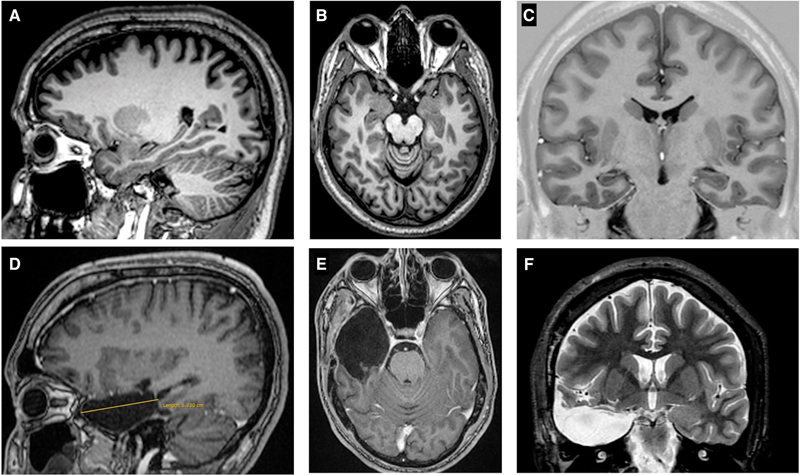
MRI (Coronal, axial and sagittal) pre and postoperative of surgery with Anterior temporal lobectomy for right hippocampal sclerosis. ( **A, B, C** ) Preoperative MRI showing right hippocampal sclerosis. ( **D, E, F** ) Postoperative MRI showing excision of the neocortex (3,5 cm) and mesial structures.

**Figure 2:**
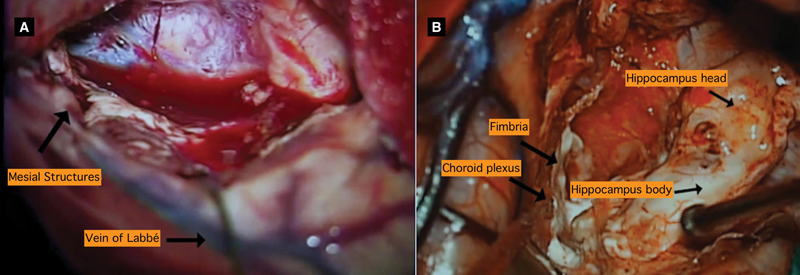
Surgical photos of anterior temporal lobectomy for right hippocampal sclerosis. ( **A** ) Surgical view after anterior temporal lobectomy. Mesial structures not yet resected. ( **B** ) Resection of mesial structures. Hippocampus and parahippocampal gyrus already disconnected.

**Figure 3:**
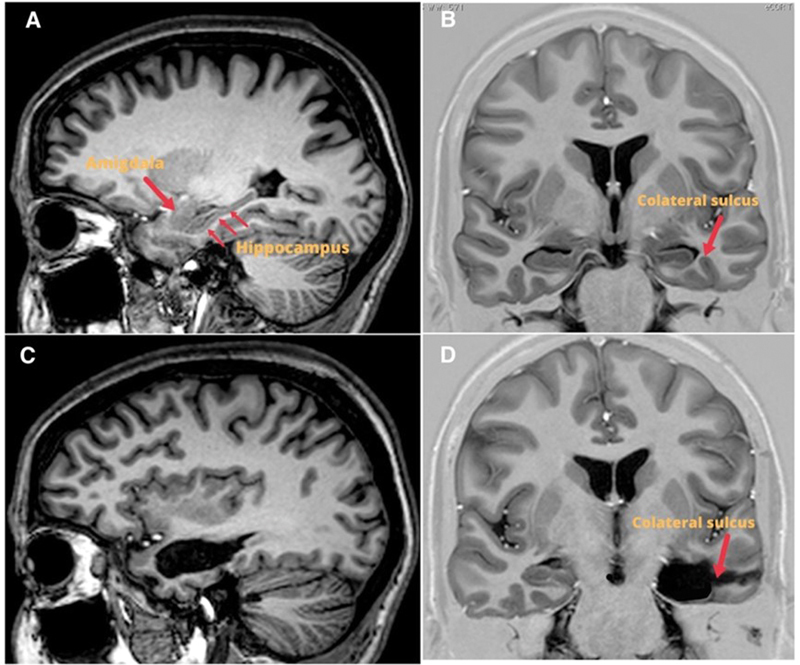
MRI (Coronal IR and sagittal T1 volumetric) pre and postoperative of surgery with selective access for left hippocampal sclerosis. ( **A** ) Preoperative coronal MRI. Collateral groove shows limit of resection of mesial structures. ( **B** ) Preoperative sagittal MRI. arrows show the hippocampus and amygdala. ( **C and D** ) Details of the resection of the mesial structures in coronal and sagittal sections.

**Figure 4:**
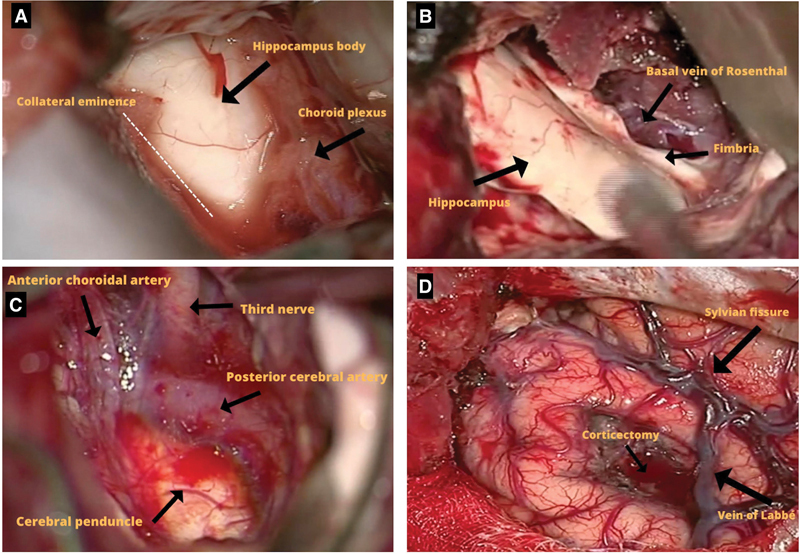
Surgical photos of selective access for left hippocampal sclerosis. ( **A** ) Initial view of the hippocampus after corticectomy. In dotted lines, the collateral eminence, lateral limit of the resection of the mesial structures. ( **B** ) After lateral disconnection at the level of the collateral and medial eminence in the ambient cistern, we visualize the basal vein of Rosenthal and the fimbria. ( **C** ) After resection of the mesial structures, we visualize the vessels of the peduncular cistern, third nerve and peduncle protected by the arachnoidal plane. ( **D** ) final view with the corticectomy area.

 The same surgeon performed all surgeries. In right-sided hippocampal sclerosis, a temporal lobectomy and hippocampectomy was performed using the Spencer technique, 12 resecting at least 3.5 cm of the anterior border of the temporal lobe ( Figures 1 and 2 ). In left hippocampal sclerosis, a selective amygdalohippocampectomy was used as described by Niemeyer ( Figures 3 and 4 ). 13

The surgical procedure of hippocampal resection is accompanied by the removal of other mesial structures, including the uncus, amygdala, and parahippocampal gyrus. The resection of the hippocampus and parahippocampal gyrus should be performed as posteriorly as possible, extending at least up to the level of the lateral mesencephalic sulcus.

### The rationale for the choice of surgical access

The choice between ATL and SAH based on the sclerosis side followed the following rationale:

- All patients have typical temporal lobe seizures;
- The Video-EEG showed seizures with semiology and a typical electrographic pattern;
- Volumetric MRI showed no lesions other than unilateral hippocampal sclerosis.

In Brazil and several other developing countries, the cost of performing surgeries is often a major obstacle. The use of invasive research to individualize access points based on electrophysiological details can significantly increase expenses, rendering procedures impossible to perform. The cost of public healthcare for the evaluation and surgical treatment of temporal lobe epilepsy, including medical fees and hospital costs, is roughly equivalent to 1200 US dollars. In this context, the addition of extensive invasive monitoring is not feasible.

On the other hand, there is a chronic shortage of epilepsy medication in the public health system, leading to uncertain clinical treatment. This further underscores the value of safe surgical interventions that can produce positive outcomes, even in the context of limited resources.

 The literature shows that both accesses have excellent results in seizure control and neuropsychological outcome. 16 17 Despite overall good results, some studies show a worse language performance in patients operated with left ATL. Similarly, SAH would have a worse outcome in epilepsy control. 17 18 22 23 24

### Statistical analysis

We utilized contingency tables to compare the two groups, analyzing the results in relation to the type of surgery performed (ATL or SAH). Additionally, we conducted a Kaplan-Meier survival analysis to examine the occurrence of seizures, the patient's ILAE 1 status, and good outcome (ILAE 1 to 3).

The data were analyzed with the IBM SPSS Statistics Software. A p- value of less than 0.05 was considered significant.

## RESULTS

Our database included 146 patients who underwent surgery for temporal lobe epilepsy secondary to hippocampal sclerosis between 2008 and 2019. Thirteen patients were excluded from our initial analysis due to incomplete medical records or follow-up loss. One patient died from pulmonary thromboembolism in the immediate postoperative period, resulting in a mortality rate of 0.06% and their exclusion from the analysis. The remaining 132 patients, comprising 72 females (53%) and 60 males (47%), were evaluated. The mean age at the time of surgery was 37.85 years (range: 9–65 years).

 Hippocampal sclerosis was present on the right side in 70 patients (53%) and on the left side in 62 patients (47%). All the surgical cases underwent anatomopathological and immunohistochemical analysis of the resected tissues, which revealed no abnormalities or neuronal depopulation suggestive of hippocampal sclerosis. There was no statistical difference between age, sex, and follow-up time in the two groups (ATL and SAH), as shown in Table 2 . The mean follow-up time after surgery was 57.2 months (range: 12–137 months). 

**Table 2:** Frequencies comparing anterior temporal lobectomy (ATL) and selective amygdalo hippocampectomy (SAH) groups for the treatment of hippocampal sclerosis (HS)

		Total	ATL Right HS	SAH Left HS	p
n		132	70 (53.0%)	62 (47.0%)	
Gender	M	60 (47.0%)	30 (42.9%)	30 (48.4%)	
	F	72 (53.0%)	40 (57.1%)	32 (51.6%)	
Mean age in years		37.85	36.94	38.87	
Mean follow up in months		57.2	55.7	58.8	
ILAE	1	58 (43.9%)	40 (57.1%)	18 (31.0%)	*p* = 0.001
	2	2 (1.5%)	0 (0.0%)	2 (3.2%)	
	3	45 (34.1%)	22 (31.4%)	23 (37.1%)	
	1 to 3*	105 (79.5%)	62 (88.6%)	43 (69.3%)	*p* = 0.006
	4	16 (12.1%)	5 (7.1%)	11 (17.7%)	
	5	11 (8.3%)	3 (4.3%)	8 (12.9%)	
	6	0 (0.0%)	0 (0.0%)	0 (0.0%)	

Note: * ILAE 1 to 3 are considered as good result.

 In the follow-up period, 66 patients (50%) had at least one seizure (excluding events within the first 30 days after surgery). At the end of the follow-up period, 105 patients (79.5%) had achieved an ILAE score of 1–3, indicating a good result. Of the patients who underwent ATL, 62 (88.6%) obtained a good result, compared with 43 (69.3%) in the SAH group ( *p*  = 0.006). Only 58 patients (43.9%) were on ILAE 1 at the end of the follow-up period, with 40 (57.1%) in the ATL group and 18 (31.0%) in the SAH group ( *p* = 0.001), as summarized in Table 2 . 

**Figure 5:**
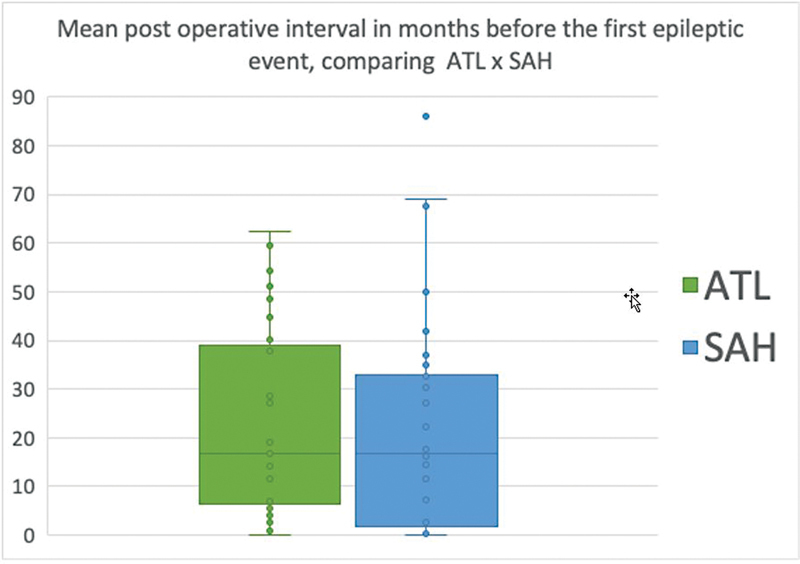
Abbreviations: ATL, anterior temporal lobectomy; SAH, selective amygdalohippocampectomy. Notes: Student *t* -test between surgery groups: *p* = 0.82. ATL mean 23.04 months; SAH mean 21.86 months. Event free interval comparing the ATL and SAH groups.

 The average interval until an epileptic event was 22.8 months (range: 1–86.1 months). Patients who underwent ATL had a mean time to the first seizure of 23.04 months, compared with 21.86 months in those who underwent SAH, with no statistical difference ( *p* = 0.82), as shown in Figure 5 . 

**Figure 6:**
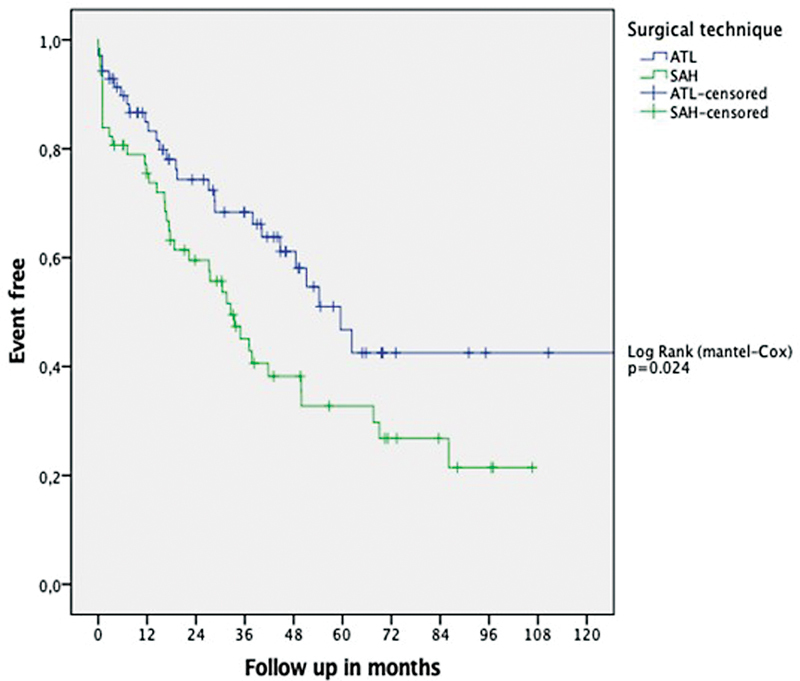
Abbreviations: ATL, anterior temporal lobectomy; SAH, selective amygdalohippocampectomy Statistical analysis using Mantel-Cox Log Rank. *p* = 0.024. Kaplan-Meier survival analysis with seizure as event, comparing ATL and SAH.

 The Kaplan-Meier mortality curves ( Figure 6 ), using the Log Rank (Mantel-Cox) statistical analysis with seizure as the event, showed a significant difference between the ATL and SAH groups ( *p*  = 0.024). 

 Our surgical morbidity rate was 11.8% (17/143), and mortality was 0.6% (1/143), consistent with the results described in the literature. The patient who died in the first postoperative week due to PTE was excluded from the analysis of results regarding epilepsy control. Other complications are summarized in Table 3 . 

**Table 3:** Complications reported in our case series

Age, sex	Complication
29, Male	Cardiorespiratory arrest in anesthetic induction
41, Male	Wound infection, cranioplasty, chronic headache
25, Female	Wound infection
43, Female	Wound infection
20, Female	Wound infection
20, Female	Wound infection
40, Female	Memory impairment
41, Female	Memory impairment
30, Female	Memory impairment
48, Male	CSF leak submitted to external lumbar shunt, acute subdural hematoma, decompressive craniectomy, wound infection, debridement, cranioplasty.
46, Female	Postoperative hemiparesis with subsequent recovery
48, Female	Visual field disorder
41, Female	Visual field disorder
51, Female	Visual field disorder
27, Male	Visual field disorder
65, Male	Intraparenchymal hematoma
39, Female	Deep vein thrombosis

Abbreviation: CSF, cerebrospinal fluid.

## DISCUSSION

 The primary purpose of surgery is to control seizures. Maintaining a good functional status of patients is also mandatory. The search for a more selective resection is based on not worsening memory and language deficits, especially in the dominant hemisphere. It has been shown in several articles that the selective resection of mesial structures has a benefit, even if marginal, in the cognitive assessment of patients. 24 25 26 27 Other authors do not show differences between ATL and SAH regarding neuropsychological prognosis. 27 28 Helmstaedter 23 postulates that the cognitive deficit after eloquent temporal resection could be more linked to perioperative cortical injury, visible in post-surgical MRI, than to the type of resection. 

 The only multicenter randomized study showing the outcome of surgery in terms of seizure control was performed by Wiebe et al. in 2001. In the group of patients operated on, always by ATL, 38% were completely free of seizures (Engel 1). Concerning the control of epilepsy, there are variable results in the literature when comparing SAH and ATL. Several authors show equality in seizure control, 29 30 31 32 while others show better results in ATL. 18 19 Clusmann, 19 in a series of 89 children and adolescents, found a worse result in seizure control in SAH when compared with ATL. Also, patients with left hippocampal sclerosis had a worse result despite the surgical technique. In a meta-analysis including 13 studies and 1203 patients, Josephson 19 found better control of seizures in ATL than in SAH. 

 Our evaluation sought to compare the two types of surgery, not only in terms of good surgical outcomes but also in the occurrence of seizures and the complete control of seizures (ILAE 1). The statistical analysis show a better outcome in occurrence of seizures ( *p*  = 0.005), ILAE 1 final result ( *p*  = 0.001) and good result ( *p* = 0.006) in favor of ATL. We performed Kaplan Meyer's survival analysis having a seizure as the target event. The curves showed better results in ATL over SAH. The Log Rank statistical analysis significantly favors the ATL group ( *p*  = 0.029). The Kaplan Meier graphs show a downward curve with a progressive worsening of the results over time, consistent with the findings described in the literature. The results are consistent with a better surgical prognosis in resections that include the temporal neocortex over more selective resections. 

 Interestingly, the interval until the first epileptic event was similar when comparing the two groups. However, patients in the SAH group had seizures more often after this initial event ( Figure 5 ). This finding could be related to the maintenance of an altered neuronal network in patients with more selective resection, which quickly resumes the pattern of seizures after a first ictal event. 22 The same difficulty of resources that makes it difficult to carry out a more individualized approach to cases makes the surgery attractive from a cost-benefit point of view. 

As the procedure is safe with very low morbidity and mortality, surgery proves to be an effective procedure for our reality. Clinical treatment has a significant cost and, in most cases, it is paid for by the state, which cannot maintain this treatment without interruptions due to lack of funds.

### Limitations of the study

 We did not assess neuropsychological, speech, and language differences between groups, as all surgeries in the left hemisphere were SAH. In our series, preoperative tests to determine hemispheric dominance for language were not performed. By convention, all cases on the left (predominantly dominant hemisphere in humans 33 34 ) were submitted to SAH, and those on the right to ATL, the interpretation of our results is limited. It is essential to mention that Clusmann, 19 in 2004, observed that surgeries in the dominant hemisphere had worse results despite the technique used. It is a possible bias in our work. Despite these limitations, we believe that the data obtained adds relevance to the discussion about the difference in results regarding the technique used. 

We excluded all patients who had MRI lesions other than HS to avoid a worse result in selective surgeries for patients who had lesions in the temporal neocortex. However, MRI is not always able to clearly show small dysplastic lesions.

The postoperative evaluation time was long enough to show the differences between the two techniques. As there is a tendency for the results to progressively worsen over time, as demonstrated in our survival curves, a longer follow-up perhaps showed that the two techniques tend to match up with a longer follow-up.

In conclusion, there is still controversy about the influence of more selective procedures in surgery for ATL epilepsy. Our work has shown better results concerning the control of epilepsy when we use ATL compared with SAH. Despite the varied results of articles on the subject, our data show that performing ATL may be more effective in controlling epilepsy, emphasizing the importance of assessing language and memory before and after surgery to define the standard of comparison between the two surgical techniques. Whenever possible, the choice of access route should be made individually for each patient, based on neurophysiological and imaging findings. In countries with a cost limitation for extended propaedeutics, ATL may be the best option for the proper control of seizures with minimal additional morbidity.

## References

[1] Mohan M Keller S Nicolson A The long-term outcomes of epilepsy surgery PLoS One 2018 13 05 e0196274 10.1371/journal.pone.0196274 29768433 PMC5955551

[2] Cascino G D Temporal lobe epilepsy is a progressive neurologic disorder: Time means neurons! Neurology 2009 72 20 1718 1719 10.1212/wnl.0b013e3181a4e465 19321843

[3] Shahani L Cervenka G Impact of surgical intervention on seizure and psychiatric symptoms in patients with temporal lobe epilepsy BMJ Case Rep 2019 12 07 e229242 10.1136/bcr-2019-229242 PMC6663174 31352381

[4] Engel J. Jr PT. (1998).Epilepsy: A Comprehensive Textbook. 2nd edArchives of Neurology.:Philadelphia Lippincott Williams & Wilkins 20081373–1374 pp. 10.1001/archneur.55.10.1373

[5] Pascual M R Temporal lobe epilepsy: clinical semiology and neurophysiological studies Semin Ultrasound CT MR 2007 28 06 416 423 10.1053/j.sult.2007.09.004 18074998

[6] Effectiveness and Efficiency of Surgery for Temporal Lobe Epilepsy Study Group Wiebe S Blume W T Girvin J P Eliasziw M A randomized, controlled trial of surgery for temporal-lobe epilepsy N Engl J Med 2001 345 05 311 318 11484687 10.1056/NEJM200108023450501

[7] Costa B S Santos M CV Rosa D V Schutze M Miranda D M Romano-Silva M A Automated evaluation of hippocampal subfields volumes in mesial temporal lobe epilepsy and its relationship to the surgical outcome Epilepsy Res 2019 154 152 156 10.1016/j.eplepsyres.2019.05.011 31153103

[8] Rosa D V Rezende V B Costa B S Circulating CD4 and CD8 T cells expressing pro-inflammatory cytokines in a cohort of mesial temporal lobe epilepsy patients with hippocampal sclerosis Epilepsy Res 2016 120 1 6 10.1016/j.eplepsyres.2015.11.011 26709876

[9] Feindel W Leblanc R de Almeida A N Epilepsy surgery: historical highlights 1909-2009 Epilepsia 2009 50 03 131 151 10.1111/j.1528-1167.2009.02043.x 19298436

[10] Hill D Falconer M A Pampiglione G Liddell D W Discussion on the surgery of temporal lobe epilepsy Proc R Soc Med 1953 46 11 965 976 10.1177/003591575304601112 13112252 PMC1918680

[11] Morris A A Temporal lobectomy with removal of uncus, hippocampus, and amygdala; results for psychomotor epilepsy three to nine years after operation AMA Arch Neurol Psychiatry 1956 76 05 479 496 13371963 10.1001/archneurpsyc.1956.02330290023003

[12] Spencer D D Spencer S S Mattson R H Williamson P D Novelly R A Access to the posterior medial temporal lobe structures in the surgical treatment of temporal lobe epilepsy Neurosurgery 1984 15 05 667 671 10.1097/00006123-198411000-00005 6504282

[13] Niemeyer P. (1958). The transventricular amygdalohippocampectomy in temporal lobe epilepsy. Temporal Lobe Epilepsy

[14] Wieser H G Yaşargil M G Selective amygdalohippocampectomy as a surgical treatment of mesiobasal limbic epilepsy Surg Neurol 1982 17 06 445 457 10.1016/s0090-3019(82)80016-5 7112377

[15] Mansouri A Fallah A McAndrews M P Neurocognitive and Seizure Outcomes of Selective Amygdalohippocampectomy versus Anterior Temporal Lobectomy for Mesial Temporal Lobe Epilepsy Epilepsy Res Treat 2014 2014(306382):306382 10.1155/2014/306382 25349728 PMC4198822

[16] Schramm J Temporal lobe epilepsy surgery and the quest for optimal extent of resection: a review Epilepsia 2008 49 08 1296 1307 10.1111/j.1528-1167.2008.01604.x 18410360

[17] Moshir Estekhareh S S Saghebdoust S Zare R Hakak M A Hashemabadi B AG Memory and executive functioning outcomes of selective amygdalohippocampectomy in patients with hippocampal sclerosis: A preliminary study in a developing country Surg Neurol Int 2022 13 161 10.25259/SNI_49_2022 35509560 PMC9062935

[18] Bate H Eldridge P Varma T Wieshmann U C The seizure outcome after amygdalohippocampectomy and temporal lobectomy Eur J Neurol 2007 14 01 90 94 10.1111/j.1468-1331.2006.01565.x 17222120

[19] Clusmann H Kral T Gleissner U Analysis of different types of resection for pediatric patients with temporal lobe epilepsy Neurosurgery 2004 54 04 847 859, discussion 859–860 10.1227/01.neu.0000114141.37640.37 15046650

[20] Commission on Neurosurgery of the International League Against Epilepsy (ILAE) Wieser H G Blume W T Fish D ILAE Commission Report. Proposal for a new classification of outcome with respect to epileptic seizures following epilepsy surgery Epilepsia 2001 42 02 282 286 11240604

[21] Omisade A O'Grady C Sadler R M Divergence between functional magnetic resonance imaging and clinical indicators of language dominance in preoperative language mapping Hum Brain Mapp 2020 41 14 3867 3877 10.1002/hbm.25092 32519808 PMC7469800

[22] Josephson C B Dykeman J Fiest K M Systematic review and meta-analysis of standard vs selective temporal lobe epilepsy surgery Neurology 2013 80 18 1669 1676 10.1212/wnl.0b013e3182904f82 23553475

[23] Helmstaedter C Van Roost D Clusmann H Urbach H Elger C E Schramm J Collateral brain damage, a potential source of cognitive impairment after selective surgery for control of mesial temporal lobe epilepsy J Neurol Neurosurg Psychiatry 2004 75 02 323 326 14742620 PMC1738880

[24] Clusmann H Schramm J Kral T Prognostic factors and outcome after different types of resection for temporal lobe epilepsy J Neurosurg 2002 97 05 1131 1141 10.3171/jns.2002.97.5.1131 12450036

[25] Rydenhag B Silander H C Complications of epilepsy surgery after 654 procedures in Sweden, September 1990-1995: a multicenter study based on the Swedish National Epilepsy Surgery Register Neurosurgery 2001 49 01 51 56, discussion 56–57. Doi: 10.1227/00006123-200107000-00007 11440459 10.1097/00006123-200107000-00007

[26] Helmstaedter C Reuber M Elger C C Interaction of cognitive aging and memory deficits related to epilepsy surgery Ann Neurol 2002 52 01 89 94 10.1002/ana.10260 12112052

[27] Roessler K Kasper B S Shawarba J Operative variations in temporal lobe epilepsy surgery and seizure and memory outcome in 226 patients suffering from hippocampal sclerosis Neurol Res 2021 43 11 884 893 10.1080/01616412.2021.1942407 34156329

[28] Nascimento F A Gatto L A Silvado C Mäder-Joaquim M J Moro M S Araujo J C Anterior temporal lobectomy versus selective amygdalohippocampectomy in patients with mesial temporal lobe epilepsy Arq Neuropsiquiatr 2016 74 01 35 43 10.1590/0004-282X20150188 26690840

[29] Wolf R L Ivnik R J Hirschorn K A Sharbrough F W Cascino G D Marsh W R Neurocognitive efficiency following left temporal lobectomy: standard versus limited resection J Neurosurg 1993 79 01 76 83 10.3171/jns.1993.79.1.0076 8315472

[30] Paglioli E Palmini A Portuguez M Seizure and memory outcome following temporal lobe surgery: selective compared with nonselective approaches for hippocampal sclerosis J Neurosurg 2006 104 01 70 78 10.3171/jns.2006.104.1.70 16509149

[31] Arruda F Cendes F Andermann F Mesial atrophy and outcome after amygdalohippocampectomy or temporal lobe removal Ann Neurol 1996 40 03 446 450 10.1002/ana.410400314 8797534

[32] Tanriverdi T Olivier A Cognitive changes after unilateral cortico-amygdalohippocampectomy unilateral selective-amygdalohippocampectomy mesial temporal lobe epilepsy Turk Neurosurg 2007 17 02 91 99 17935023

[33] Carey D P Johnstone L T Quantifying cerebral asymmetries for language in dextrals and adextrals with random-effects meta analysis Front Psychol 2014 5(1128):1128 10.3389/fpsyg.2014.01128 25408673 PMC4219560

[34] Tzourio N Crivello F Mellet E Nkanga-Ngila B Mazoyer B Functional anatomy of dominance for speech comprehension in left handers vs right handers Neuroimage 1998 8 01 1 16 10.1006/nimg.1998.0343 9698571

